# The Bubonic Plague

**Published:** 1897-02

**Authors:** 


### THE BUBONIC PLAGUE.

Recent dispatches from London say that two cases of the bubonic plague have developed there—the same disease which since 1894 has been ravaging certain parts of China, says the *New York World.* This is only another reminder to us here in America that these Eastern epidemics only too frequently girdle the earth before they have run their course, as did la grippe, which first was heard from in an alarming form in distant Russia, but which soon reached over our shores with the disastrous results which are still so fresh in mind.

We are exposed on two sides to invasion by the bubonic plague, which is nothing more nor less than the "black death," which in 1665 killed 100,000 Englishmen and produced the London horrors De Foe so graphically describes. It may reach us by the Golden Gate from China and it may come by any one of our Atlantic ports should the disease really become rooted in Europe. Already two cases of it have occurred on one of the Pacific Mail steamers on the way from China to San Francisco. Two Chinese stokers were stricken and died of the disease shortly after the vessel sailed, and both were buried at sea.

It is said on good authority that the bubonic plague has been more or less prevalent in certain parts of China ever since the seventeenth century, when it gained such terrific headway that it swept all Europe and nearly depopulated Marseilles, to say nothing of the De Foe horrors it wrought in London. The disease took pestilential form in Canton about two years ago. There is a wide, open ditch about a portion of the city into which all the refuse and exuvia of the swarming population is emptied. It is the placid Chinese theory that the tide washes the festering mass away. The tide does nothing of the kind, and the filth remains there in the blistering sun to the depth of two or three feet, a breeding place for myriads of rats and other foul vermin.

It is a curious fact that in all violent outbreaks of the bubonic plague rats seemed first to be seized with it, and their death in great numbers always preceded by a few days corresponding ravages among human beings. The rats swarming out of the extra-mural ditch about Canton and dying by thousands was the first warning that the Chinamen had that an epidemic of the disease was upon them. They burned red paper, tried to fool the gods by changing the date of the new year, and died by the thousand convinced that they had taken all the sanitary precautions within the power of man.

From China the disease got to Formosa and the Malay Peninsula. The Japanese, with their modern learning and freedom from the idiotic superstitions which afflict the besotted Chinamen, made a strong and intelligent fight against the deadly invader and kept him comparatively at bay. But the disease crept into India, and among certain groups of the natives there has spread with frightful rapidity. A press dispatch from Bombay last week reported 2,094 cases and 1,494 deaths up to date. Europeans and such natives as were able to get away were leaving the city by thousands. The newspapers were threatening martial law unless the natives obeyed the sanitary regulations.

In India, as in China, the advent of the plague was heralded by an epidemic among rats, which were found dead by the hundreds in and about the native dwellings. In some instances rats, evidently suffering from the disease, swarmed, swollen and dying, into rooms where were human beings. They reeled and staggered and wandered aimlessly about as though in the same delirium which marks a certain stage of the disease in man.

A feature of the disease is the suddenness of attack and the awful rapidity with which one stage follows another until death ensues. The first symptom is usually a chill, as in ague. Then follows an acute nervousness and mental disturbance, with a fever that sends the temperature up from 100 to 107. The patient staggers like a drunken man. Headache, a burning thirst and intense pain in the upper part of the abdomen follow.

A sticky perspiration exudes from the pores, and then follows the glandular swellings from which the disease takes its name.

These last occur in the groin or neck, or, very frequently, under the armpits. The swelling is oval and egg-like in shape, and the more of them there are the less dangerous is the attack. Sudden, stab-like pains shoot through the body, and this gave rise to the superstition among the Turks that the man with the plague is wounded by the arrow of an invisible devil.

Dark spots—whence comes the name of the "black death"—appear on the skin of the victim just before dissolution. These spots were called "the token," and their appearance was the signal of abandonment of all hope in the Middle Ages, and the victim was then and there scared to death by being told that all was over.

An eminent Japanese bacteriologist, Prof. Kita-Soto, who studied in Europe under Koch, has discovered the microbe of the "black death," and his discovery was confirmed by Prof. Gersin, formerly attached to the Pasteur Laboratory in Paris. The bacillus is short, thick, easy of culture, and when inoculated on guinea pigs, kills them in twenty-four hours. Specimens of bacilli-infected glandular swellings taken from victims of the disease, have been forwarded to Paris, and it is hoped a vaccine may be obtained that will prove efficacious.—*The Public Health Journal, January*, 1897.

